# Habituation in Predictability-Modulations of Stimulus-Response Binding

**DOI:** 10.5334/joc.438

**Published:** 2025-03-10

**Authors:** Philip Schmalbrock, Jan Theeuwes, Christian Frings

**Affiliations:** 1 Department of Psychology, University of Trier, DE; 2 Institute for Cognitive & Affective Neuroscience (ICAN), University of Trier, DE; 3 Vrije Universiteit Amsterdam, NL

**Keywords:** Action Control, S-R binding effect, Predictability, Statistical Regularities, Habituation

## Abstract

Acting intentionally requires the integration of perceptual with action information in a common representational format. In the action control literature, this integrated representation is often called event file and is measured in so-called stimulus-response binding effects. These effects allow us to measure the strength of this shared representation and the impact it can have on behavior. A well-established finding is that particular variables can modulate the size of binding effects – one recently discovered modulator is *stimulus predictability*: If perceptual information is perfectly predictable, stimulus-response binding effects diminish. Yet, the concrete mechanism of why predictability diminishes stimulus-response binding effects remained elusive so far. In the present study (*N* = 234), we compared two possible explanations for these modulation effects, namely habituation versus statistical learning. We found that it is unlikely that the predictability modulation is explained by habituation. Instead, we found evidence that is more in line with (but not exclusive to) learning of statistical regularities as an explanation. Our study thus adds to recent attempts to more closely relate learning mechanisms and action control.

## Statement of Significance

As humans, we often must ignore irrelevant information in our surroundings. Yet, although we achieve this with remarkable ease, this irrelevant information can still affect our mental processes. Specifically, it has been found that irrelevant information can strongly influence our actions. Recently, it has been found that this is not the case if the irrelevant information repeats multiple times and becomes predictable. We ask here which mechanisms underlie this observation. It turns out, (at least partially) we rely on learning of regularities to achieve this attenuation of irrelevant information for action control.

## Introduction

Our everyday life is full of different actions. Some are simple, like grabbing and pulling the door handle. Others are complex, like playing a piece of music on an instrument. Although it is often assumed that complex actions also require complex processes to achieve them, most people are less aware that simple actions also need the same complex interplay between different cognitive processes (Hommel, 2009; see also Moravec, 1995). The literature investigating this interplay is often called action control. Action control research has focused on feature-binding processes that assumably allow us to combine inputs from perceptual sources and motor processes into one coherent representation – regardless of the actions’ complexity.

Specifically, the action control literature assumes that perceptual information (e.g., color or form; but see Münster & Frings, 2025) and action information (e.g., the direction of an action or the hand used; but see Nemeth et al., 2024) are together *integrated* into a short-lived episodic memory trace (Hommel et al., 2001), called *event file* (Hommel, 2004). This event file can be thought of as a momentary interconnection between all features deemed relevant and necessary for an action. Imagine it like this: You need to bring together the information about the shape, location, and maybe the orientation of an object to adequately choose arm extension, grip strength, and execution speed. Presumably, this is possible due to a common representational format that treats action and perceptual features the same way (Prinz, 1997).

Importantly, if shortly after the event file creation some or all features contained in an event file repeat, this previously created event file is *retrieved or re-activated*. This is possible because the previous event file is maintained for a short time (Hommel & Frings, 2020): Features in an event file remain interconnected for a small amount of time after the action is executed. Yet, these features may be used differently in a present action episode and effortful processes are needed to dissolve the previous interconnections (cf. Stoet & Hommel, 1999). Note that this strict separation of integration and retrieval into two distinct processes has only recently been emphasized in the literature (cf. Frings et al., 2020; Beste et al., 2023) but has been backed by a broad consensus in the action control literature (Frings et al., 2024).

Importantly, retrieved event files can influence actions in the present episode. Depending on the degree of overlap between past and present, performance costs and benefits can emerge. Compared to episodes with no overlap, repeating all features in an event file (e.g., response *and* stimulus) introduces performance benefits while, conversely, behavioral costs are introduced if only some features repeat (e.g., response *or* stimulus; cf. Hommel, 1998; Frings et al., 2007). The former is likely happening because the previous event file can be effortlessly reused (Henson et al., 2014). The latter is likely happening because additional required processes become activated that resolve the mismatch between the previously created event file and the present episode (Henson et al., 2014). Together, the literature often describes these costs and benefits as S(timulus)-R(esponse) binding effects.

Importantly, different variables can modulate S-R binding effects. That is, costs and benefits can be reduced (Schmalbrock & Frings, 2022), increased (Schmalbrock et al., 2022), or can even be abolished (Schmalbrock et al., 2023). One suggested mechanism behind modulation is intentional weighting (Memelink & Hommel, 2013). In a nutshell, each feature is assigned a priority value (i.e., feature weight, Zelinsky & Bisley, 2015 or Narhi-Martinez et al., 2023 for an overview) that determines the importance for the cognitive system to process this specific feature. The intentional weighting account postulates that these feature weights are used to determine whether a feature is considered for action control processes like integration into an event file or retrieval from it.^1^ Imagine it like this: to adapt optimally to our environment, our brain should select only the information of the highest relevance which requires a weighting system that decides what is relevant and what is irrelevant.

One modulator that has recently been found is *stimulus predictability* (Schmalbrock et al., 2023; but see also Frings et al., 2019). That is, if participants can predict what an upcoming stimulus will be, they do not show the typically observed S-R binding effects.^2^ Specifically, by repeating the same stimulus over and over again, participants could anticipate with absolute certainty what an upcoming stimulus would be (while they never knew which response they would give). In these previous studies, the stimulus bound to a response was not even task-relevant. This is important because S-R binding mechanisms are so reliable that even irrelevant stimuli yield S-R binding effects (cf. Frings et al., 2007). Thus, our cognitive system usually processes even these irrelevant stimuli. Yet, it tunes out irrelevant information if constantly repeated (although it is unclear if these stimuli are not processed at all or only kept out of action control processes).

### Mechanisms of Predictability

However, it is unclear what the specific mechanism that results in this modulating effect is. In our previous study (Schmalbrock et al., 2023) we reasoned that predictability modulations for S-R binding effects emerge due to the learning of statistical regularities (cf. Wang & Theeuwes, 2018a, 2018b, 2018c). That is, our cognitive system learns that a) distractors are irrelevant to the main task and b) that these irrelevant distractors never change. Thus, they hold no information value for responding at all. Therefore, we can safely reduce the processing of these distractors because we learned that there is no need to divert many processing resources toward them.

This would likely be achieved by reducing the strength of the priority signal that signals the relevance of a specific stimulus to our cognitive systems. These priority signals function as a kind of processing resource managing information: High values indicate a large need for allocating processing resources, low values indicate a neglectable need for allocating processing resources (see Theeuwes & Failing, 2020 or Narhi-Martinez et al., 2023 for extensive reviews). Specifically, if a stimulus has a naturally high processing priority, our cognitive system can artificially reduce the signal. Thus, reducing the likelihood of this stimulus being processed at all. There exist actual neural correlates of these priority signals termed priority maps that have been found in many areas of the cortex but are highly relevant for early, visual processing (Zelinsky & Bisley, 2015; cf. Treisman & Gelade, 1980).

Predictability modulations thus may emerge because our cognitive system is *de-prioritizing* the signals produced by a repeatedly presented, irrelevant distractor after it has been produced. That distractors in S-R binding tasks are very early modulated has recently been shown using early, sensory EEG correlates of lateralized presented distractors (Pastötter & Frings, 2018). We term this account the de-prioritization account as this highlights the actual process that is taking place (compared to learning of statistical regularities that focuses on general perception).

Yet, a habituation-based approach to our previous data may also explain the patterns observed in our previous study.^3^ Habituation is a phenomenon where our cognitive system or even single cells become less responsive to stimulation after repeated exposure (Thompson & Spencer, 1966; see also Sokolov, 1963). Specifically, if repeatedly presented, the signal produced by a stimulus is already weak even before it gets properly processed (compared to the signal produced by unrepeated stimuli). This decrease in responsiveness increases with more frequent presentations (Rankin et al., 2009). Habituation plays a central role in several aspects of learning theories (e.g., in Pavlovian conditioning, Wagner & Rescorla, 1972; operant conditioning, McSweeney et al., 1996; evaluative conditioning, De Houwer et al., 2001).

Habituation constitutes a very early process of perceptual learning that relies heavily on *constant stimulus repetition* to be maintained. However, learning of statistical regularities is a flexible learning mechanism relying on the *recurrence of patterns*. Although seemingly very similar, both require different forms of periodicity for learning to take place and stick in place. For habituation, even a single violation of the constant repetition is enough to interfere with the reduced responsiveness (Rankin et al., 2009). Conversely, learning of statistical regularities outlives even a few violations of a constant repetition as long as there is a general rule that can be followed (e.g., Wang & Theeuwes, 2018a, 2018b, 2018c).

For action control, it is quite important to differentiate between these forms of learning. Previous research and theorizing highlighted the stark similarity between action control and learning processes in general (cf. Frings et al., 2023). Through repeated exposure to specific stimulus and response pairings, an ever-strengthening binding emerges that is – after enough repetitions – transferred from short- to long-term memory. Thus, it is quite essential for this line of research to better understand what types of learning processes (dis-)allow the transfer from short- to long-term bindings and how different forms of learning interact.

Yet, our previous study (Schmalbrock et al., 2023, especially Experiment 1) is not fit for further differentiating which learning processes are causing the predictability-based modulating effects. The focus of our previous study was the difference this manipulation makes for integration and retrieval but not the reason why these effects emerged in the first place. In our previous line of research, we ran three experiments, of which two focused on the question of how predictability affects integration and/or retrieval. Our manipulation was either only applied to the integration process or the retrieval process. Our results showed that only the retrieval process was actually affected by this manipulation. That is, less stimulus-response binding costs and benefits emerged here (compared to a control condition). Nonetheless, *learning* resulted in the absence of S-R binding effects. Therefore, it is essential to better understand these modulation effects to better understand the interrelation between action control and learning.

Here this can easily be achieved by a rather distinct feature of habituation: its *dishabituation* (see Rankin et al., 2009). Specifically, if the repeated stimulation with the same stimulus is withheld and interrupted by another stimulus, responsiveness to the habituated stimulus is quickly restored.^4^ This is different from de-prioritization-based approaches that also work when the stimulation is withheld or interrupted (see e.g., Wang & Theeuwes, 2018a, 2018b, 2018c where the typical setup is never perfectly predictable, i.e., regularly interrupted).

In our previous study, we used only a maximized predictability setup (Schmalbrock et al., 2023; see Frings et al., 2019 for the same problem). That is, the exposure to the repeating distractor was never interrupted by other stimuli. Thus, it was not possible to observe dishabituation and, therefore, differentiate between de-prioritization (unaffected by repetition interruption) and habituation (affected by repetition interruption). As dishabituation is a distinct feature of habituation but not of de-prioritization, an optimal paradigm introduces a chance for habituation. If habituation plays a role in the previously observed modulation of S-R binding effects (Schmalbrock et al., 2023), then allowing dishabituation to occur should yield stronger S-R binding effects compared to a condition that does not allow dishabituation. If habituation plays no significant role in this previous modulation, introducing a chance for dishabituation should yield the same costs and benefits in a condition with habituation allowed versus a condition without habituation allowed.

### The Present Study

To investigate the role of habituation in predictability effects,^5^ we used the same experimental design as in our previous study (Schmalbrock et al., 2023). That is, participants worked through a Distractor-Response Binding paradigm (Frings et al., 2007). In this paradigm, participants must respond to a central target letter, flanked by two identical distractor letters. Crucially, a single trial consists of two separate displays that each require a response. A first prime and a second probe display. Thus, we can introduce a relationship between prime and probe displays that generates conditions fit for investigating S-R costs and benefits.

That is, in some trials response and distractor repeat from prime to probe, resulting in performance benefits (full repetition). In other trials, either response or distractor repeats but the other feature changes, resulting in performance costs (partial repetition). Lastly, in some trials, neither response nor distractor repeat, resulting in neither costs nor benefits (full change). Note that this setup allows us to generate just the condition under which we would assume repetition costs and benefits to emerge. That is, a complete, partial, and no overlap between a past and a present action episode.

In our previous study (Schmalbrock et al., 2023), we created two groups of participants. One group worked through this standard setup of the paradigm (low predictability), while a second group worked through several blocks of this paradigm (maximized predictability). Importantly, the blocks in the second group presented perfectly predictable distractor stimuli. For example, in the first block participants only saw the letter D as prime and probe distractors, while in the second block, they always saw the letter K as prime distractor and the letter D as probe distractor. Note that this also means that the relation between prime and probe distractor is also always fixed (i.e., always repetition or always change; see below). All the while the prime/probe target, that determined the response, changed unpredictably. Thus, each block also generated a specific, fixed prime-probe relation for the distractor, while the prime-probe relation for the response varied unpredictably.

Yet, in this previous study, predictability per block was maximized: Participants could predict perfectly what the next distractor would be (i.e., there was no deviation from the rule). As pointed out above, this makes it impossible to disentangle habituation and de-prioritization explanations, as it does not allow for dishabituation to emerge. We, therefore, introduced a third experimental group. This third group worked through the same setup as the maximized group. However, predictability in their blocks was not maximized as it was routinely interrupted by an unpredicted distractor letter (e.g., a K instead of D in an otherwise all-D block). This manipulation was simultaneously applied to the prime and the probe distractors (see also Figure 2). We termed this group the *increased* predictability group as prediction is still possible (compared to the low predictability group) but less certain (compared to the maximized predictability group).

This difference between increased and maximized predictability groups only applied to the distractor identity. The contingency between prime-to-probe relations for distractors remained the same between both groups. Further, because the prime-probe relation for distractors also remained fixed in a block we can solely focus on the question of habituation by directly comparing the maximized and increased predictability group. That is, the only difference between the two groups is the regular interruption of the distractor identity but never the distractor relation.

We thus compared the S-R binding effect in this increased predictability group to the S-R binding effects in the maximized predictability group (repetition is absolutely certain), where the repeated exposure was never interrupted by other stimuli, and the *low* predictability group where the upcoming distractors were not predictable.

**Figure 1:**
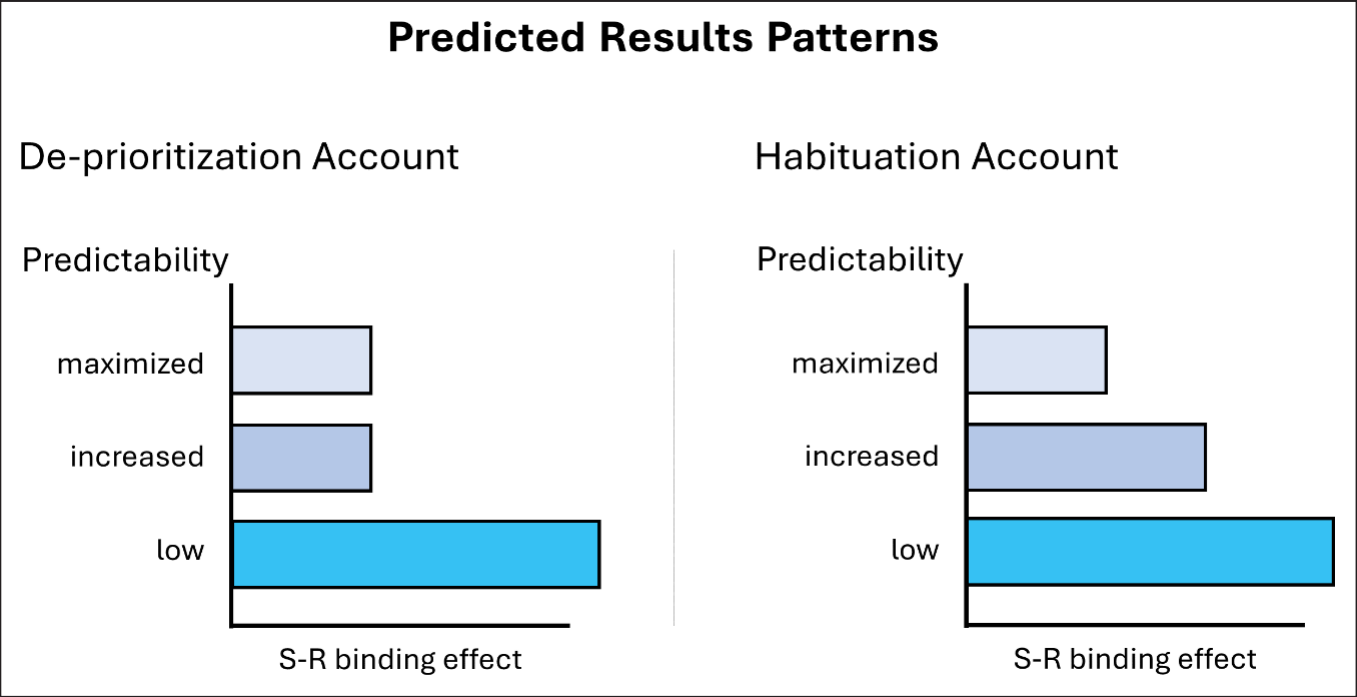
Expected Result Patterns for the De-prioritization Account and the Habituation Account. *Note*. The De-Prioritization Account suggests no difference between maximized and increased predictability groups. The Habituation Account suggests weaker S-R binding effects in the maximized compared to the increased predictability groups.

The modulation effect should weaken/disappear in the increased predictability group if habituation plays a role (see Figure 1, right panel). Due to dishabituation, distractors should be more strongly processed compared to the maximized predictability group. However, there should be no difference between increased and maximized groups if habituation plays no role at all (see Figure 1, left panel). This would be also in line with the de-prioritization account. Additionally, we should observe a strong reduction in S-R binding effects in the maximized predictability group and a strong S-R binding effect in the low predictability group (i.e., a replication of our previous results, Schmalbrock et al., 2023). These results should be observable in a significant three-way interaction (between response relation, distractor relation, and predictability) in a mixed-effects analysis of variance on either reaction times and/or error rates and complementary post-hoc *t*-tests on the S-R binding effects computed on the respective dependent variable. Note here, that for the increased predictability condition, we only analyzed the trials that followed the rule of a block (i.e., the common trials but an analysis focusing only on the rare trials, that yields the same results, can be found in the **Supplementary Material;** an analysis of the first, second, and third trial after a rare trial can also be found in the **Supplementary Material**, it did not yield any difference for these trials either).^6^

**Figure 2:**
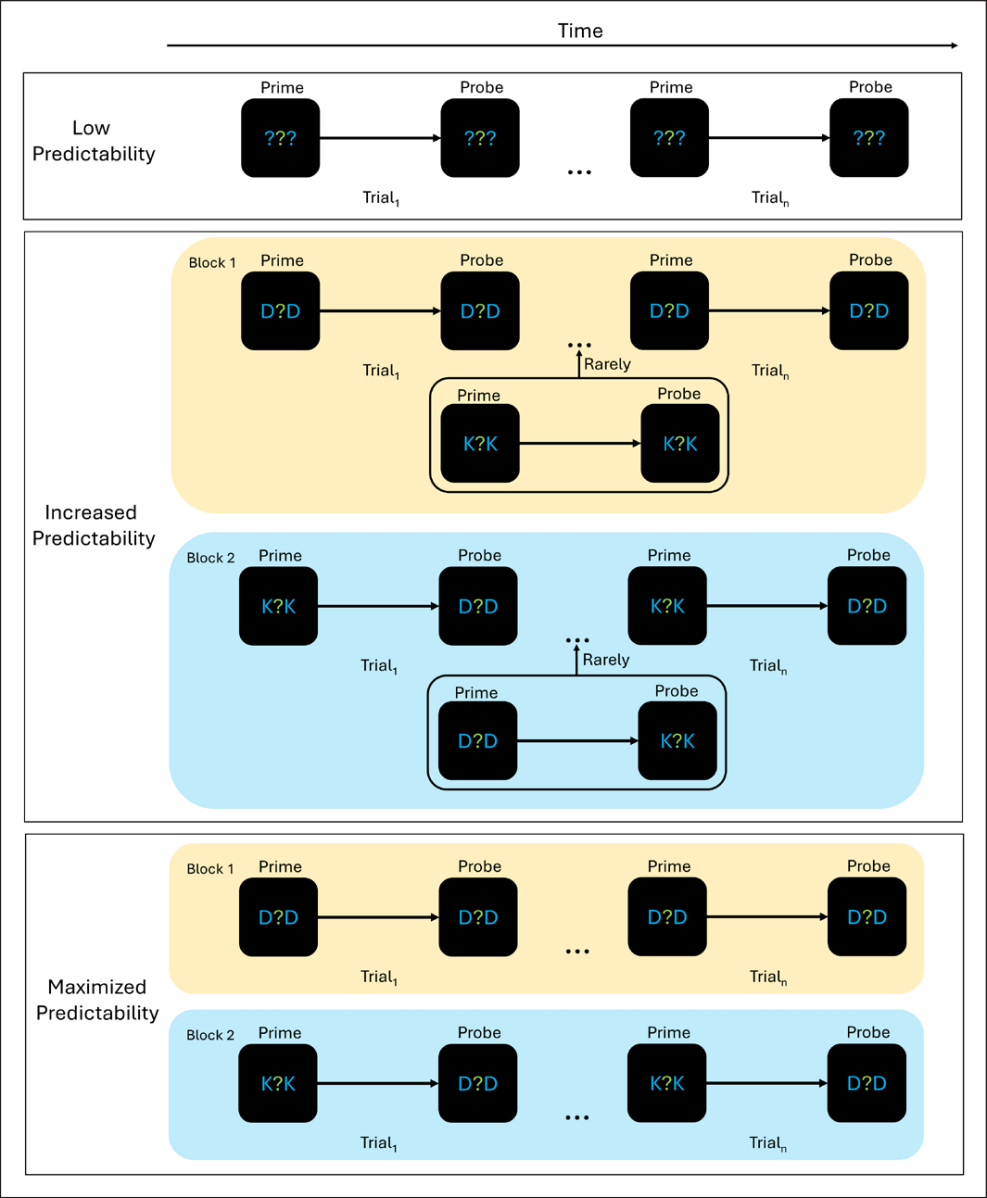
Experimental logic with three different experimental groups. *Note*. Experimental setup. Participants identified the centrally presented, green letter. To them, it was always unpredictable which response they would have to give. Presentation of the different experimental trial types was trial-wise in the low predictability group and blocked in both the increased and maximized groups. Within these blocks, the presentation of the two blue distractors followed a strict rule. Prime and probe distractors never changed within a block in the maximized group. In the increased group, prime and probe distractors were mostly not changed but rarely other distractors were shown. The four different configurations for prime and probe distractors were: Prime D, Probe D; Prime K, Probe K; Prime K, Probe D; Prime D, Probe K. For readability purposes, only two exemplary blocks are presented here. In total, there were four blocks in the increased and maximized predictability group.

## Experiment

### Methods

#### Participants

240 students from Trier University participated.^7^ The final sample consisted of 234 participants (190 female, 43 male, 1 diverse; 215 right-handed) with a median age of 22 years because the data of 6 participants was excluded/lost. We lost the data of two participants due to a technical error (1 in the low predictability group and 1 in the increased group). Datasets of four participants were excluded because they were outliers in either RT (both low group) or error rate (one maximized group and one low group) according to the taxonomy by Tukey (1977). For their 0.5h of service, participants received partial course credits. The ethical standards set by the Trier University administration were followed for this study. The sample size was calculated according to previous studies investigating the S-R binding effect, which typically led to medium-sized effects (*d* = 0.35 to 0.5; cf. first study using the basic paradigm, Frings et al., 2007 or our previous paper on the topic of S-R binding effects and predictability, Schmalbrock et al., 2023). Thus, we planned to run at least *N* = 70 participants in each of the three groups, leading to a power of at least 1 – *β* = .80 (assuming an α = .05; GPower 3.1.9.2; Faul et al., 2007), to observe the basic S-R binding effect in all groups (assuming a rather small S-R binding effects of *d* = 0.35) without pinpointing the size of the assumed interaction. Due to the online nature of our experiment, we anticipated a larger drop-out compared to offline experiments. Thus, we recruited an additional 10 participants per group. In total, we sampled 240 participants (80 per group).

Considering that the largest number of participants we lost in a group was 3 (N = 77 in the low group) the smallest basic distractor-response binding effect size we were able to detect was *d* = 0.32 with α = .05 and 1 – β = .80.

#### Design

Our experimental setup manipulated two within-participant factors and one between-participant factor: response relation (within: response repetition vs. response change), distractor relation (within: distractor repetition vs. distractor change), and distractor predictability (between: low vs. increased vs. maximized).

#### Apparatus & Stimuli

We programmed the experiment in Psychopy (Peirce et al., 2019; Version 03.01.2020) and ran it online via Pavlovia (Peirce & MacAskill, 2018). There were two relevant and distinct displays (see Figure 2): a first prime and a second probe display. In each of the two displays, we presented participants with a string of three letters at the screen center (Font: Arial; Font size 25 pixels). The central letter was presented in green (RGB_255_: 0, 128, 0) which marked it as the target for the participants. Letters used as the target were ‘J’ and ‘F’ (both presented equally often as prime and probe targets). The two irrelevant, flanking letters were presented in blue (RGB_255_: 0, 0, 128) which marked them as the distractor letters. For these distractors, the letters ‘D’ or ‘K’ were used (both presented equally often as prime and probe distractors but see Procedure). Throughout the experiment, a black background was used (RGB_255_: 0, 0, 0).

#### Procedure

Participants signed up for participation via the recruitment platform used at Trier University (Sona Systems; sona-systems.com) and received a link that directed them to the experiment on pavlovia.com. In the instructions, participants were tasked with placing their left index finger on the ‘F’ key and their right index finger on the ‘J’ key. We emphasized that they should respond as fast and as correctly as possible. Before the experiment started, participants had to work through 12 training trials with random pairings of responses and distractors. Here, participants received performance feedback after each prime and each probe display (For a correct response: “Correct!”; For a wrong response: “Wrong!”; both in German language). In the experiment proper, participants received feedback only after erroneous responses (“Wrong! Please respond as fast and correct as possible”; in German).

The task consisted of two consecutive responses. In both, prime and probe displays, participants had to classify the identity of the centrally presented, green target letter. If the letter was an F, they responded with the ‘F’-key; if the letter was a ‘J’, they responded with a ‘J’-key press.

In total, the experiment proper consisted of 384 trials. Every 24 trials, participants could take a self-paced break. A single trial (i.e., a prime and a probe display) consisted of the following chain of events: The start of a trial was marked by the presentation of a fixation mark (+) presented at the screen center for 1000 ms. After this, the prime display was presented. The display ended after participants made a response or if the display duration of 1000 ms had elapsed. Prime and probe were separated by a blank screen presented for 500 ms.^8^ Similar to the prime display, the probe display was presented until participants made a response or 2000 ms elapsed. As our main dependent variable was probe reaction time, we increased the response window in the probe to ensure that we lost fewer trials through response omission. 1500 ms passed before a new trial started.

We varied the factors response relation and distractor relation orthogonally. For response repetition trials, the prime response was repeated in the probe. Vice versa, for response change trials, a different response was required in the probe compared to the prime. For distractor repetition trials, prime and probe distractors were identical. For distractor change trials, prime and probe distractors always differed. Note that this orthogonal manipulation means that the prime display is not indicative of the probe display (but see the next paragraph).

The factor predictability was varied as a between-participant factor. That is, participants in the low predictability group worked through one long block (384 trials) of a standard S-R binding task. Specifically, the target, the distractors, and the prime-to-probe relation varied unpredictably for them. In the maximized predictability group, participants worked through four short blocks (96 trials each) of the same task but with a fixed distractor type in the prime/probe and a fixed prime-to-probe relation between the distractors. In the increased predictability group, participants worked through the same setup as the maximized predictability group. However, in ~17% of all trials in a block, the predictable distractors were replaced with untypical distractors (i.e., in an only ‘D’s block, a ‘K’ was presented instead). This manipulation was applied to prime and probe distractors simultaneously. It was contingent on the prime-probe relation. That is, for example, in a Prime K, Probe D block, the distractors were changed to Prime D, Probe K. Rare distractor letters were randomly inserted into the trial stream. Note that the predictability manipulation only applied to the distractor identity and the distractor prime-to-probe relation. The target/response and the response prime-to-probe relation varied unpredictably. The four different configurations for prime and probe distractors were: Prime D, Probe D; Prime K, Probe K; Prime K, Probe D; Prime D, Probe K (see also Table 1).

**Table 1:** Overview Trial combinations.


PREDICTABILITY	GROUP	BLOCK 1	BLOCK 2	BLOCK 3	BLOCK 4

LOW	NO GROUPS	NO BLOCKS			

Increased	A	D (Prime), D (Probe)	K, K	D, K	K, D

	B	D, D	K, D	D, K	K, K

	C	D, K	K, D	D, D	K, K

	D	D, K	K, K	D, D	K, D

Maximized	A	D, D	K, K	D, K	K, D

	B	D, D	K, D	D, K	K, K

	C	D, K	K, D	D, D	K, K

	D	D, K	K, K	D, D	K, D


*Note*. In the low predictability group, no blocks but a single stream of trials was presented. Hence neither different groups nor blocks existed.

#### Transparency and openness

We report how we determined our sample size, all data exclusions (if any), all manipulations, and all measures in the study, and we follow JARS (Appelbaum et al., 2018). All data (raw and analysis) are available at [https://doi.org/10.23668/psycharchives.14467]. The data was collected from end of 2022 to the start of 2024. Data were analyzed using R, version 4.0.4 (R Core Team, 2018), and the package ‘dplyr’ (Wickham et al., 2021) was used for data processing and aggregation. This study’s design and its analysis were not pre-registered.

## Results

To compute the S-R binding effect, we calculated the distractor repetition benefit in response repetition trials minus the distractor repetition cost in response change trials.^9^ Note that calculating a S-R binding effect simplifies the analysis processes because simple *t*-tests can be calculated, instead of an ANOVA + post-hoc tests. The S-R binding effect essentially breaks down the two-way interaction Response Relation × Distractor Relation (that signifies S-R binding effects and yields four different values) for a given dependent variable into a single value.

Experimental conditions were first compared using a mixed-effect analysis of variance (ANOVA) with type-III sums of square, using the ‘ezAnova’-function from the package ‘ez’ (Lawrence, 2016). S-R binding effects in the different groups were compared using Welch’s *t*-tests (see Delacre et al., 2017). These post-hoc *t*-tests were corrected for α-error accumulation using the method by Bonferroni (1936). That is, the *adjusted* two-sided significance level for the Welch’s *t*-test comparing groups is *α* = .017 \[
[\alpha = {\textstyle{{significance\;level} \over {number\;of\;comparisons}}} = {\textstyle{{.05} \over 3}} = .017]
\] .

We supplemented all of our *t*-tests with Bayesian *t*-tests because one of our hypotheses rested on a non-significant difference. These used a standard Cauchy prior with \[
{\rm rscale} = {\textstyle{{\sqrt 2} \over 2}}
\] (Rouder et al., 2009; cf. Jarosz & Wiley, 2014 for interpretation). The 95% confidence intervals we report are for the mean difference.

### Data Processing

For all analyses of the increased predictability group, only trials following the general rule of the block were analyzed as these trials were the target of our predictability manipulation. We chose this approach because we thought this to be the most appropriate way to make the maximized and increased groups comparable. We thus removed every trial from the analysis where the distractor was not the distractor typically displayed in this experimental block^10^ (this only applies to the increased predictability group).

Further, we considered only probe trials for our analysis where prime responses were correct. Additionally, only trials with RTs longer than 200 ms and shorter than 1.5 interquartile ranges over the third quartile (before probe error exclusion) of each person’s RT distribution were analyzed (see Tukey, 1977). That is, 4% of all trials were excluded due to these two constraints. For the analysis of probe RT, all trials with wrong probe responses were also excluded, that is an additional 10% of all trials (14% in total).

### Reaction Times

**Figure 3:**
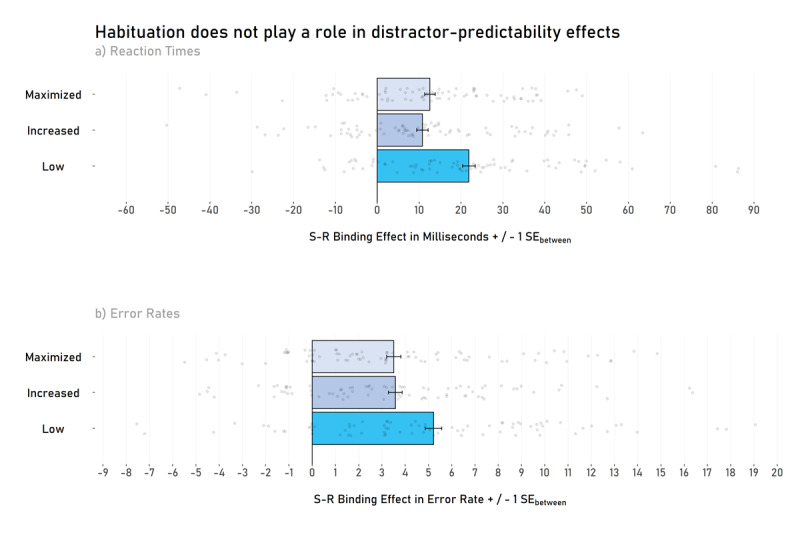
Stimulus-Response binding effects as a function of predictability (Low vs. Increased vs. Maximized). Panel **a)** shows S-R binding effects in reaction times and panel **b)** shows S-R binding effects in error rates. *Note*. There is no difference in S-R binding effects between the maximized and increased predictability groups in reaction times (Panel a) or error rates (Panel B). However, there is a difference between maximized/increased groups and the low predictability group (at least statistically in the reaction times and descriptively in both reaction times and error rates). Transparent dots represent the individual S-R binding effect of each participant. Error bars show between-participants standard errors.

**Figure 4:**
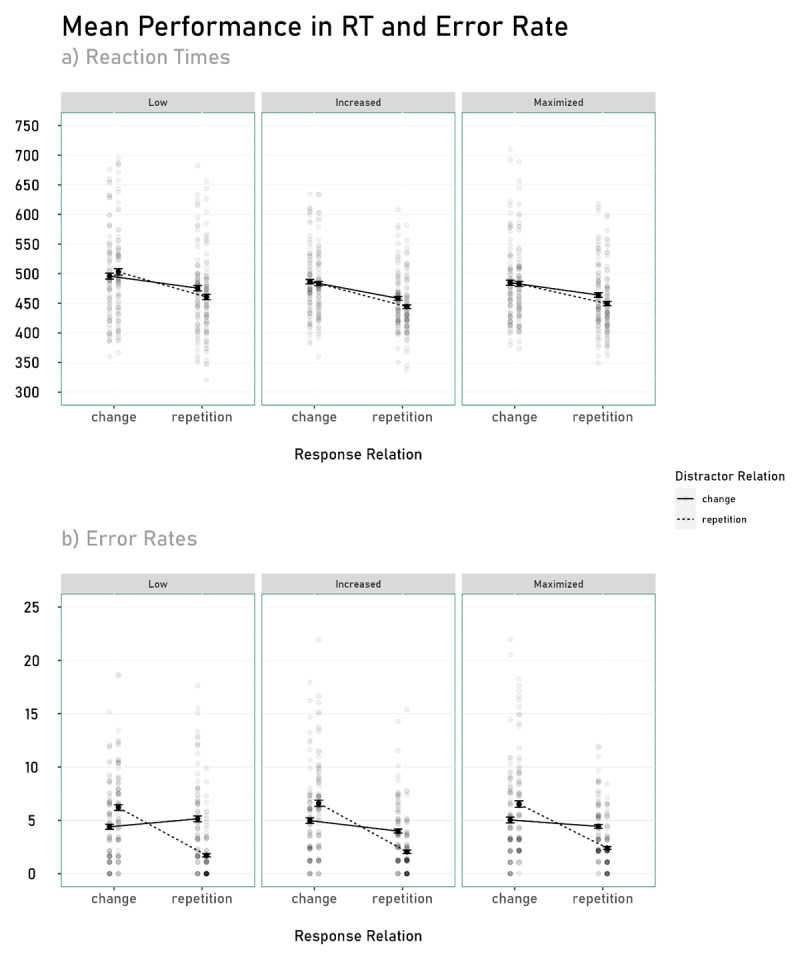
Mean Reaction Times and Error Rate as a function of Response Relation, Distractor Relation, and Predictability. *Note*. Transparent dots represent the individual performance of each participant. Error bars show between-participants standard errors.

A 2 (response relation: repetition vs. change; within) × 2 (distractor relation: repetition vs. change; within) × 3 (predictability: maximized, increased vs. low; between) mixed-effects ANOVA on probe RTs yielded a significant interaction between response relation and distractor relation, *F*(1, 230) = 119.10, *p* < .001, *η_P_*^2^ = .34, indicating significant S-R binding effects. Intriguingly, this interaction was further modulated (i.e., there was a three-way interaction) by predictability, *F*(2, 230) = 6.12, *p* = .003, *η_P_*^2^ = .05, suggesting that the S-R binding effect is modulated by the level of predictability (see Figures 3a and 4a).

This three-way interaction is further supplemented by Welch’s two-sample *t*-tests (with a significance level corrected to *α* = .017): S-R binding effects in the low predictability group (*M* = 22 ms, *SD* = 23) significantly differed from the increased predictability group (*M* = 11 ms, *SD* = 21), *t*(148.95) = 3.13, *p* = .002, *d* = 0.20, *BF*_10_ = 14.29, 95% CI [4.08, 18.10], and the maximized predictability group (*M* = 13 ms, *SD* = 19), *t*(145.57) = 3.21, *p* = .008, *d* = 0.18, *BF*_10_ = 5.00, 95% CI [2.52, 16.10]. There was no significant difference between the maximized and the increased predictability group, *t*(153.89) = 0.56, *p* = .578, *d* = 0.04, *BF*_01_ = 5.03, 95% CI [–4.52, 8.07].

S-R binding effects differed from zero in the low predictability group, *t*(75) = 8.20, *p* < .001, *d* = 0.94, *BF*_10_ > 1000, 95% CI [16.57, 27.19], in the increased predictability group, *t*(77) = 4.61, *p* < .001, *d* = 0.52 *BF*_10_ > 1000, 95% CI [6.13, 15.49], and in the maximized predictability group, *t*(78) = 5.81, *p* < .001, *d* = 0.65, *BF*_10_ > 1000, 95% CI [8.26, 16.87].

Additionally, a main effect for response relation emerged, *F*(1, 230) = 309.68, *p* < .001, *η_P_*^2^ = .57. Participants responded faster in response repetition trials (M = 459 ms, SD = 58) compared to response change trials (M = 489 ms, SD = 67). Also a main effect for distractor relation emerged, *F*(1, 230) = 39.21, *p* < .001, *η_P_*^2^ = .15. Participants responded faster in distractor repetition trials (M = 470 ms, SD = 66) compared to distractor change trials (M = 477 ms, SD = 63). No further main effect or interaction reached significance, all *F*s < 1.81 and *p*s > .16.

### Error Rates

A 2 (response relation: repetition vs. change; within) × 2 (distractor relation: repetition vs. change; within) × 3 (predictability: maximized, increased vs. low; between) mixed-effects ANOVA on probe Error Rates yielded a significant interaction between response relation and distractor relation, *F*(1, 230) = 166.06, *p* < .001, *η_P_*^2^ = .42, indicating significant S-R binding effects. This interaction was further modulated by predictability, *F*(2, 230) = 3.06, *p* = .049, *η_P_*^2^ = .03, suggesting that the S-R binding effect was modulated by the level of predictability (see Figures 3b and 4b).

This effect is, however, not further supplemented by Welch’s two-sample *t*-tests (with a significance level corrected for α-error accumulation of α = .017): S-R binding effects in the low predictability group (*M* = 5% ms, *SD* = 5) did not significantly differ from the increased predictability group (*M* = 4%, *SD* = 4), *t*(145.27) = 2.05, *p* = .042, *d* = 0.13, *BF*_10_ = 1.20, 95% CI [0.06, 3.23], and did not significantly differ from the maximized predictability group (*M* = 4%, *SD* = 5), *t*(147.80) = 2.10, *p* = .037, *d* = 0.14, *BF*_10_ = 1.32, 95% CI [0.10, 3.32]. There was no evidence of a significant difference between the maximized and the increased predictability group, *t*(157.87) = –0.09, *p* = .929, *d* < 0.01, *BF*_01_ = 5.79, 95% CI [–1.50, 1.38].

S-R binding effects differed from zero in the low predictability group, *t*(75) = 8.41, *p* < .001, *d* = 0.96, *BF*_10_ > 1000, 95% CI [3.98, 6.46], in the increased predictability group, *t*(78) = 7.07, *p* < .001, *d* = 0.75, *BF*_10_ > 1000, 95% CI [2.47, 4.55], and in the maximized predictability con group, *t*(77) = 6.70, *p* = .001, *d* = 0.75, *BF*_10_ > 1000, 95% CI [2.57, 4.58].

Additionally, a main effect for response relation emerged, *F*(1, 230) = 62.58, *p* < .001, *η_P_*^2^ = .29. Participants made fewer errors in response repetition trials (M = 3%, SD = 3) compared to response change trials (M = 6%, SD = 5). Also a main effect for distractor relation emerged, *F*(1, 230) = 9.33, *p* = .003, *η_P_*^2^ = .06. Participants made fewer errors in distractor repetition trials (M = 4.37%, SD = 4.35) compared to distractor change trials (M = 4.85%, SD = 4.05). No further main effect or interaction reached significance, all *F*s < 1.85 and *p*s > .160.

## General Discussion

The present study tried to determine which learning mechanism drives predictability effects in action control (see e.g., Schmalbrock et al., 2023). In this previous study, we could show that predictability reduces S-R binding effects. However, it remained unclear what the specific mechanism behind this reduction is. Due to action controls’ close association with learning in general, it was essential to determine what type of learning drives modulation by predictability. Our present experimental setup allowed us to gain insight into the contributing factors of learning of regularities and habituation to these modulation effects.

Essentially, we found evidence not in line with the habituation account. We found a modulating influence of predictability on S-R binding effects. Yet, effects in the maximized and increased groups did not differ, but both did differ significantly from the low group.^11^ This pattern is incompatible with the habituation account, as this account would assume a significant difference between increased and maximized groups. Instead, our results are more compatible with a de-prioritization account that assumes no difference between maximized and increased groups.

We must acknowledge that our study cannot offer an exhaustive explanation of why predictability effects emerge in action control. What we can offer is evidence that speaks against habituation as the main contributor to these effects, and evidence being in line with the de-prioritization account while other mechanism might also contribute to these effects (e.g., stimulus variability, Schmalbrock et al., 2022 or desensitization, Davison, 1968).

Further, our results also replicate and specify the results from our previous study (Schmalbrock et al., 2023): Predictable distractors *reduce* S-R binding effects but *do not* eliminate them. In our previous study, we found no S-R binding effects in the maximized predictability group. In contrast to this, in the present study, we found strong (RT: *d* = 0.65) and reliable (RT: *BF*_10_ = 100) S-R binding effects in the maximized predictability group. This difference likely emerged due to the difference in prime-probe-interval. In our previous studies, we used a prime-probe-interval of 1000 ms; in our present study, we used a 500 ms interval. The longer interval likely led to stronger event file decay (cf. Hommel & Frings, 2020) before the probe response could be given and thus diminished the influence the retrieved event file could have on probe performance.

Furthermore, our results fit well with the de-prioritization account. Regular interruption does not affect the predictability modulation in the increased predictability group. In general, this fits well with broader theoretical frameworks that assume a (de-)prioritization based on learned regularities (e.g., Awh et al., 2012). This is not to say that learning of regularities is the only driver of the effects we observed, our results just align with it. We might speculate here that if learning of statistical regularities is the main process driving these modulations, it is also unclear precisely when this prioritization would take place in the present study.

On the one hand, (de-)prioritization might take place at an early selective stage which decides whether a stimulus is processed any further (see e.g., Theeuwes & Failing, 2020). That is, the early processing priority for highly predictable but irrelevant stimuli is changed so that they are not selected for further processing at all. If a stimulus is not processed in the beginning, it is not available for later processes either (e.g., Narhi-Matinez et al., 2024; Won et al., 2022). Specifically, they would not be available for integration/retrieval.

On the other hand, (de-)prioritization might take place at a later more output (action) focused stage which decides if a stimulus is considered for action control (e.g., Memelink & Hommel, 2013; Theeuwes et al., 2024). That is, learned regularities determine whether it is more likely to execute a previously integrated and then retrieved response or the presently required response. In the present study, a response integrated with the repeatedly presented distractor should be less relevant to the system altogether. Thus, reencountering it should reduce the distractors’ ability to start retrieval.

Alternatively, these accounts do not have to be mutually exclusive: information about the relevance of distractors might be used not for only one but many processes. As Narhi-Martinez et al. (2023) point out, selective processes likely take place multiple times throughout processing. That is, depending on the context and the present processing step information is prioritized differently. This account is not only well in line with previous studies (see Zelinsky & Bisley, 2015 for a review) but would also solve the problem of situating (de-)prioritization in the present study: information about stimulus repetitions would be used for attentional selection *and* action control processes as these processes work in tandem to achieve the common goal of acting efficiently – an assumption well in line with attention (Hommel et al., 2019) and action control research (Frings et al., 2020).

What is likely is that based on our previous research (Schmalbrock et al., 2023), our results can be mainly attributed to the retrieval process of action control (whether directly or via reduced processing due to e.g., de-prioritization). In our previous study, we applied the predictability manipulation only to the prime (integration) or the probe (retrieval). We found that only in the latter manipulation predictability effects emerged. Therefore, it would be essential for future research to further differentiate how integration/binding and retrieval are affected by the manipulation used in the present experiment, in line with the recently proposed BRAC framework (Frings et al., 2020).

Although it is beyond the scope of the present manuscript, investigating the interplay between action, perception, and learning is required to further understand how humans achieve the remarkable feat of goal-directed actions. One such suggestion is that S-R integration/retrieval is a necessary stage preceding long-term (associative) learning (see Frings et al., 2023). That is, incidental linking of stimulus and response information in working memory after an action leads to a continuous build-up of linking-strength between information upon repeated pairing that is ultimately transferred into long-term memory. If true, this would offer a holistic perspective on goal-directed action as it would comprise perceptual (stimulus), action (response), and learning processes under one framework.

In conclusion, our present study investigated the role habituation plays in predictability modulations in action control. We found no evidence in line with a habituation-based explanation for these predictability modulations. Rather, our results fit better with de-prioritization accounts that are well established in attention and action control research – highlighting the link between both fields (cf. Lamy et al., 2024).

## Data Accessibility Statement

Material and analysis code is not available. The data for the experiment is available at PsychArchives under https://doi.org/10.23668/psycharchives.14467, and none of the experiments were preregistered.

## Additional File

The additional file for this article can be found as follows:

10.5334/joc.438.s1 Supplementary Material. Additional analyses.

## Footnotes

Note, however, that the intentional weighting account is agnostic to the point in time this information is used. That is, it is unclear if these weights are actually used for action control (determining what is integrated/retrieved) or if they are only used to determine which information receives access to the limited processing resources in general.

That is, no benefits emerged for repeated stimuli + repeated responses and no costs emerged for repeated stimuli + changed responses/changed stimuli + repeated responses.

Note, this is not to indicate that habituation and learning of statistical regularities are the only processes that contribute to predictability modulations – Other processes like sensitization (Groves & Thompson, 1970) or sensitivity to distractor variability (Qiu et al., 2022; Schmalbrock et al., 2022) can of course also contribute to modulations. Here we focus on the role of habituation to determine if habituation actually contributes to these types of modulation.

The decreased responsiveness to a habituated stimulus can also be restored over time by completely withholding the habituated stimulus, a process termed spontaneous recovery (Rankin et al., 2009).

Note here that we make no claims about the specific timepoint in processing from perception to action that these processes we are interested in are enfolding. See the General Discussion for different possibilities.

We thank Iring Koch and two anonymous reviewers for suggesting this analysis.

Due to the way we recruited, our sample consisted solely of students from Trier University. However, action control effects are rather basic and general effects applying to a range of populations (cf. Münster et al., 2024).

Note this differs from our previous study (Schmalbrock et al., 2023) where we used a prime-to-probe interval of 1000 ms. We changed this in the present study as recent findings show more reliable S-R binding effects for shorter intervals (Hommel & Frings, 2020).

We calculated these effects following the procedure of Frings et al., 2007. That is used a double difference on the mean performance in the different trial types using the following formula: [Response: Repetition/Distractor: Change – Response: Repetition/Distractor: Repetition] – [Response: Change/Distractor: Change – Response: Change/Distractor: Repetition].

Please find an analysis of only these trials in the **Supplementary Material**. Note that these analyses, yield identical results to the analysis reported in the main text. That is, in both RTs and error rates a significant three-way interaction emerged between predictability, response relation, and distractor relation. Further, in both RT and error rates, there was a significant difference between the low predictability group and the maximized groups + a significant difference between the low and increased groups. There was no difference between the increased and maximized groups.

Note that this pattern only emerged significantly in the RTs. In the error rate, the pattern was descriptively the same but failed to reach significance. This divergence between these two dependent variables is not uncommon in the literature but, as of yet, there is no exhaustive explanation for this phenomenon (cf. Schöpper et al., 2022 for a brief discussion on this topic.).
