# Supplementary material for: Habituation in Predictability-Modulations of Stimulus-Response Binding
Source: J Cogn. 2025 Mar 10;8(1):27. doi: 10.5334/joc.438 (PMC11931097; doi:10.5334/joc.438)
Supplement: Supplementary Material. — Additional analyses. [file joc-8-1-438-s1.pdf]

Supplementary Material

The analyses presented here only include those trials in the increased predictability condition that deviated from the expected pattern in a block; everything else is identical to the analyses presented in the main text. Specifically, only those trials of the increased predictability condition are considered that present a distractor that is not usually presented in a specific block. Note that due to the rarity of these trials (i.e., ~17% of all trials in that condition), the power of the increased condition in this analysis is strongly reduced compared to the other conditions. This resulted in weaker interaction effects in the ANOVA and weaker comparison effects in the *t*-tests. However, even in these trials there is no indication of a difference between increased and maximized predictability condition.

Reaction Time

A 2 (response relation: repetition vs. change, within) x 2 (distractor relation: repetition vs. change, within) x 2 (predictability: maximized, increased vs. low, between) mixed-effects ANOVA on probe RTs yielded a significant interaction between response relation and distractor relation, $F(1, 230) = 59.42$, $p < .001$, $\eta_P{}^2 = .21$, indicating significant DRB effects. Intriguingly, this interaction was further modulated by predictability, $F(2, 230) = 3.58$, $p = .003$, $\eta_P{}^2 = .03$, suggesting that the DRB effect is modulated by the level of predictability (see **Figure 2**).

This effect is further supplemented by Welch's two-sample *t*-tests (with a significance level corrected for α-error accumulation of $p = .017$): S-R binding effects in the low predictability condition ($M = 22$ ms, $SD = 23$) significantly differed from the increased predictability condition on the one-sided level ($M = 10$ ms, $SD = 42$), $t(123.17) = 2.26$, $p = .025$, $d = 0.15$, $BF_{01} = 0.57$, 95% CI [1.51, 22.56], and the maximized predictability condition on a two-sided level ($M = 13$ ms, $SD = 19$), $t(145.57) = 3.21$, $p = .008$, $d_z = 0.18$, $BF_{01} = 0.20$, 95% CI [2.52, 16.10]. There was *no* significant difference between the maximized and the increased predictability condition, $t(109.54) = 0.54$, $p = .592$, $d_z = 0.03$, $BF_{01} = 5.08$, 95% CI [-7.35, 12.80].

S-R binding effects differed from zero in the low predictability condition, $t(75) = 8.20$, $p < .001$, $d_z = 0.94$, $BF_{01} < 0.01$, 95% CI [16.57, 27.19], in the increased predictability condition, $t(77) =$

2.14, p = .036, d_z = 0.24, BF_{01} = 0.93, 95% CI [0.68, 19.00], and in the maximized predictability condition, t(78) = 5.81, p < .001, d_z = 0.65, BF_{01} = 0.01, 95% CI [8.26, 16.87].

Additionally, a main effect for response relation emerged, F(1, 230) = 262.04, p < .001, ${\eta_P}^2$ = .53, and a main effect for distractor relation emerged, F(1, 230) =28.12, p < .001, ${\eta_P}^2$ = .11. No further main effect or interaction reached significance, all Fs < 1.69 and ps > .187.

Error Rates

A 2 (response relation: repetition vs. change, within) x 2 (distractor relation: repetition vs. change, within) x 2 (predictability: maximized, increased vs. low, between) mixed-effects ANOVA on probe Error Rates yielded a significant interaction between response relation and distractor relation, F(1, 230) = 75.08, p < .001, ${\eta_P}^2$ = .25, indicating significant DRB effects. This interaction was further modulated by predictability on the one-sided level, F(2, 230) = 2.46, p = .087, ${\eta_P}^2$ = .02, suggesting that the DRB effect was modulated by the level of predictability (see **Figure below**).

This effect is, however, not further supplemented by Welch's two-sample t-tests tests (with a significance level corrected for α-error accumulation of p = .017): S-R binding effects in the low predictability condition (M = 5 % ms, SD = 5) did not significantly differ from the increased predictability condition (M = 3 %, SD = 9), t(123.96) = 1.92, p = .057, d_z = 0.12, BF_{01} = 1.09, 95% CI [-0.07, 4.79], and did not significantly differ from the maximized predictability condition (M = 4 %, SD = 5), t(147.80) = 2.10, p = .037, d_z = 0.14, BF_{01} = 0.76, 95% CI [0.10, 3.32]. There was no evidence of a significant difference between the maximized and the increased predictability condition, t(112.55) = 0.55, p = .584, d_z = 0.04, BF_{01} = 5.05, 95% CI [-1.69, 2.99].

S-R binding effects differed from zero in the low predictability condition, t(75) = 8.41, p < .001, d_z = 0.96, BF_{01} < 0.01, 95% CI [3.98, 6.46], in the increased predictability condition, t(78) = 2.70, p = .008, d_z = 0.28, BF_{01} = 0.28, 95% CI [0.75, 4.97], and in the maximized predictability condition, t(77) = 6.70, p = .001, d_z = 0.75, BF_{01} > 0.01, 95% CI [2.57, 4.58].

Additionally, a main effect for response relation emerged, $F(1, 230) = 71.34$, $p < .001$, ${\eta_P}^2 = .24$, and a main effect for distractor relation emerged, $F(1, 230) = 11.81$, $p < .001$, ${\eta_P}^2 = .05$. No further main effect or interaction reached significance, all *F*s < 1.53 and *p*s > .219.

Figure

Stimulus-Response binding effects as a function of predictability (Low vs. Increased vs. Maximized). Panel a) shows S-R binding effects in reaction times, panel b) shows S-R binding effects in error rates.

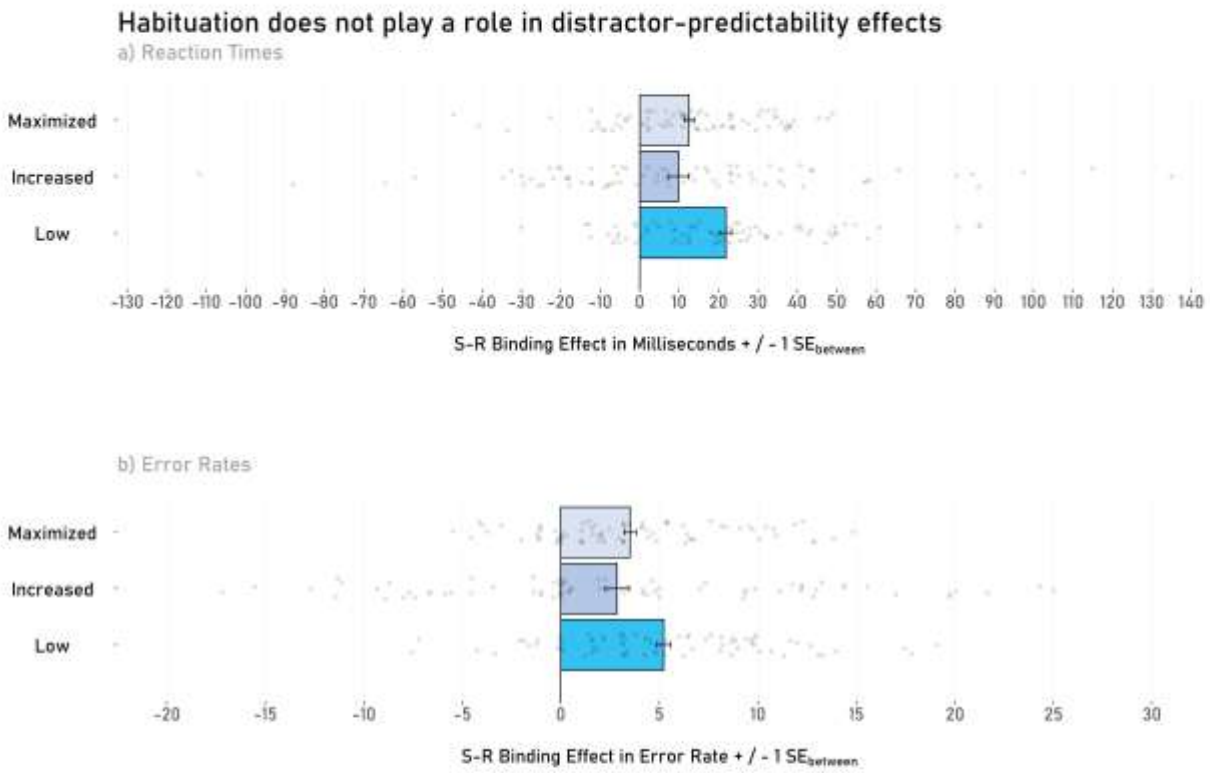

Note. Points represent the individual S-R binding effect of each participant. Error bars show between-participants standard errors.

Analysis of first, second, and third trials after rare trials

The present analysis investigates the rare trials in the increased predictability condition and their effect on the first, second, and third trials after such a rare event. Specifically, we compared how the distractor-response binding effect (IA: response relation * distractor relation) differs between common

trials in each block and the first, second, and third trial after a rare event. Neither analysis yielded any significant difference in distractor-response binding effect in either RT or error rates.

**AV**	**Trial type**	***F***	***DF***	***p***
RT	n+1	0.02	1,77	.898
	n+2	0.67	1,77	.417
	n+3	0.14	1,77	.714
Error Rate	n+1	< 0.01	1,77	.921
	n+2	1.52	1,77	.221
	n+3	0.31	1,77	.580
